# MRI markers of cerebrospinal fluid dynamics predict dementia and mediate the impact of cardiovascular risk

**DOI:** 10.1002/alz.70699

**Published:** 2025-10-23

**Authors:** Hui Hong, Yutong Chen, Dan J. Tozer, Yeerfan Jiaerken, Peiyu Huang, Hugh S. Markus

**Affiliations:** ^1^ Stroke Research Group, Department of Clinical Neurosciences University of Cambridge Cambridge UK; ^2^ Department of Radiology, School of Medicine Second Affiliated Hospital of Zhejiang University Hangzhou China

**Keywords:** cardiovascular risks, dementia, magnetic resonance imaging proxies of cerebrospinal fluid dynamics

## Abstract

**INTRODUCTION:**

Impaired cerebrospinal fluid (CSF) dynamics may contribute to dementia, but human evidence is limited. We examined associations between magnetic resonance imaging–based proxies of CSF dynamics and incident dementia, and whether CSF dysfunction mediates links between cardiovascular risk and dementia.

**METHODS:**

Using the UK Biobank, we measured CSF dynamics: perivascular space (PVS) volume, diffusion tensor image analysis along the PVS (DTI‐ALPS), blood oxygen level–dependent CSF (BOLD‐CSF) coupling, and choroid plexus (CP) volume. We assessed cardiovascular risk factors and their associations with CSF dynamics and dementia based on general practitioner, mortality, and hospital records. Mediation analysis evaluated CSF dysfunction in cardiovascular risk–dementia relationships.

**RESULTS:**

Lower DTI‐ALPS, lower BOLD‐CSF coupling, and higher CP volume predicted dementia, but PVS volume did not. DTI‐ALPS and CP volume mediated the effect of white matter hyperintensities and diabetes duration on dementia.

**DISCUSSION:**

Impaired CSF dynamics may lead to dementia and partially mediate cardiovascular risk–dementia associations.

**Highlights:**

- We developed fully automated methods for quantifying diffusion tensor image analysis along the perivascular space (DTI‐ALPS) and blood oxygen level–dependent cerebrospinal fluid (BOLD‐CSF) coupling.
- Three CSF dynamics markers—BOLD‐CSF coupling, DTI‐ALPS, and choroid plexus (CP) volume—were predictive of incident dementia, whereas PVS volume was not.
- Magnetic resonance imaging proxies of CSF dynamics markers were associated with cardiovascular injury. CP volume and DTI‐ALPS mediated the associations of both white matter hyperintensities and diabetes with dementia.

## 1 BACKGROUND

Impaired cerebrospinal fluid (CSF) dynamics have been suggested to play a key role in the development of dementia.^1^, ^2^ CSF is continuously produced by the choroid plexus (CP), generating pressure that drives its flow from the ventricular system to the subarachnoid space. From there, CSF enters the brain parenchyma via the perivascular spaces (PVSs), where it exchanges with interstitial fluid (ISF). This exchange facilitates the clearance of metabolic waste from the brain through the PVS. Impaired CSF dynamics has been demonstrated in animal models of dementia, both Alzheimer's disease (AD)^3^, ^4^ and vascular dementia.^5^, ^6^ In AD, it is suggested that this results in impaired clearance of amyloid beta (Aβ) and tau, while impaired drainage of other toxic metabolites may play a role in other dementias.^7^, ^8^ In APP/PS1 mice (a model of AD), toxic Aβ accumulation associated with impaired CSF motion along the PVS.^4^ Dysfunction of CSF dynamics preceded significant Aβ deposits, suggesting a role early in the disease process.^9^ This has led to the suggestion that impaired glymphatic function, which relies on effective CSF drainage, plays a causal role in human dementias. However, animal studies rely on invasive techniques to study CSF dynamics, such as injections of tracers into the CSF, techniques difficult to implement in humans. Therefore, there are limited data demonstrating whether CSF dynamics dysfunction contributes to dementia risk in humans.

Recently, a number of magnetic resonance imaging (MRI)‐based proxies have been proposed allowing different aspects of CSF dynamics to be non‐invasively studied in humans. These include PVS volume, diffusion tensor image analysis along the PVS (DTI‐ALPS), blood oxygen level–dependent CSF (BOLD‐CSF) coupling, and CP volume.

Enlarged PVSs are quantified by assessing their volume using T1‐weighted imaging (T1WI). Their enlargement reflects fluid stagnation within PVSs, potentially caused by diminished arterial pulsation.^10^ DTI‐ALPS uses diffusion MRI (dMRI) to assess diffusion of water molecules along PVSs that are presumed to travel along deep medullary veins.^11^ It is proposed that higher DTI‐ALPS indicates faster CSF diffusivity and therefore better of clearance function in CSF dynamics. BOLD‐CSF coupling relies on resting state functional MRI (rs‐fMRI) to simultaneously capture cerebral hemodynamic changes, and fluctuations of CSF inflow from the spinal cord into the brainstem.^12^ As hemodynamic changes in the brain drive CSF dynamics, stronger coupling indicates better CSF flow. For CP, it generates CSF and also serves as an interface for waste clearance from the brain.^13^ Increased CP volume has been associated with reduced CSF production and waste clearance.^14^ All these metrics have been demonstrated to correlate with cognitive function or dementia in recent studies.^15^, ^16^, ^17^, ^18^, ^19^, ^20^, ^21^ However, most studies have been based on small sample sizes and cross‐sectional analysis, and focused on a single marker.

A further question is what causes CSF dynamics dysfunction in humans. Vascular risk and markers of cardiovascular dysfunction have been associated with CSF dynamics dysfunction in animal studies. Multiple epidemiological studies have demonstrated cardiovascular risk factors, and markers of cardiovascular dysfunction in both the brain and heart, are risk factors for dementia.^22^, ^23^, ^24^ It has been hypothesized that cardiovascular risks contribute to the development of dementia via CSF dynamics dysfunction,^25^, ^26^ but evidence for this in humans is lacking.

To address these questions, we determined the relationship between these MRI proxies of CSF dynamics and incident dementia in a large community‐based population. We further determined relationships between the proxies of CSF dynamics MRI markers with vascular risk factors and markers of cardiovascular dysfunction, and then applied mediation analysis to investigate if cardiovascular risk increased dementia risk via CSF dynamic dysfunctions.

## 2 METHODS

### 2.1 Participants

Participants were selected from the imaging sub‐study within the UK Biobank, a longitudinal cohort of 500,000 participants. MRI had been performed in 48,880 participants at the time of the study. MRI included T1 and fluid‐attenuated inversion recovery (FLAIR), dMRI, and rs‐fMRI.^27^ Only imaging data from the first imaging visit were used. We excluded participants with corrupted image files. We additionally excluded participants with a history of AD dementia, vascular dementia, frontotemporal dementia, stroke, multiple sclerosis, Parkinson's disease, motor neuron disease, traumatic brain injury, idiopathic intracranial hypertension, normal pressure hydrocephalus, schizophrenia, autism spectrum disorder, and major depressive disorder at the time of MRI visit as these disorders were shown to affect CSF dynamics in human subjects.^28^, ^29^, ^30^, ^31^ Additionally, participants with a history of a brain tumor were excluded to avoid the impact of tumor‐related structural distortion on quantification of CSF dynamics. For T1‐based metrics, we excluded participants with the head motion in the top 1% (UK Biobank field ID 24419). For DTI‐based metrics, we additionally excluded participants with the highest mean relative head motion (UK Biobank field ID 24453) within the top 1%. For BOLD‐CSF coupling analysis, we excluded participants with fewer than five CSF‐containing voxels in the bottom three slices of the rs‐fMRI images. As the BOLD‐CSF coupling value is non‐negative, we excluded participants with BOLD‐CSF coupling value < 0. Among the remaining participants with BOLD‐CSF coupling results, we excluded participants with the highest mean relative head motion during rs‐fMRI scanning within the top 1% (UK Biobank field ID 24441). The cohort selection workflow is shown in Figure S1 in supporting information.

**RESEARCH IN CONTEXT**

1. **Systematic review**: We searched PubMed from its inception to December 1, 2024, for English‐language studies investigating the relationship between magnetic resonance imaging (MRI) markers of cerebrospinal fluid (CSF) dynamics dysfunction and the risk of developing dementia, using the terms “(CSF dynamics OR glymphatic* OR DTI‐ALPS* OR perivascular space* OR choroid plexus OR BOLD‐CSF*) AND (dementia* OR cognitive OR cognition).” Existing studies suggest that MRI markers of CSF dynamics dysfunction could serve as novel imaging indicators for dementia. However, most of these studies have been limited by small sample sizes, cross‐sectional designs, and a focus on single MRI markers. Furthermore, while cardiovascular risk is a well‐established risk factor for CSF dynamics dysfunction in animal models, there is currently limited evidence on how CSF dynamics dysfunction mediates the association between cardiovascular risk factors and dementia risk in vivo.
2. **Interpretation**: Our study developed automated methods to extract these metrics from large datasets and investigated their association with dementia risk in a community‐based cohort of 45,000 individuals. We found that three markers— blood oxygen level–dependent (BOLD)‐CSF coupling, diffusion tensor image analysis along the perivascular space (DTI‐ALPS), and choroid plexus (CP) volume—were predictive of incident dementia. Moreover, these markers may partially mediate the increased dementia risk associated with cardiovascular factors. Our findings support a role for impaired CSF dynamics as a significant pathological mechanism in the development of dementia and provide evidence that disrupted CSF dynamics may partially mediate the increased risk of dementia associated with cardiovascular factors.
3. **Future directions**: Impaired CSF dynamics is a risk factor for dementia and may partially mediate the link between cardiovascular risk and dementia. Multicenter and multiethnic studies using our open‐access automated method are needed to replicate and validate our findings.

Demographics including age at time of MRI scanning were recorded. Education was inferred (field 6138) according to a previous study.^32^ Participants with a “college or university degree” were assigned 20 years of schooling, “A levels/AS levels” 13 years, “O levels/General Certificate of Secondary Education (GCSE)” 10 years, “Certificate of Secondary Education (CSE)” 10 years, “National Vocational Qualification (NVQ) or Higher National Diploma (HND) or Higher National Certificate (HNC)” 19 years, and “other professional qualifications” 15 years.

### 2.2 Cardiovascular risk factors, and cardiac and arterial function measures

Cardiovascular risk factors, and measurement of cardiac and arterial functions, were obtained from the same visit as when the MRI took place.

Systolic blood pressure (BP) and diastolic BP were obtained from automated readings (fields 4080 and 4079). Pulse pressure was calculated from systolic BP minus diastolic BP. Hypertension status and the duration of hypertension prior to MRI visit were obtained by identifying diagnoses of essential and/or secondary hypertension at or before the imaging visit (fields 131286 and 131294, respectively). Diabetes status and the duration of having diabetes prior to MRI visit were obtained from field 2976. Smoking was quantified by the number of pack years, obtained from field 20161. Alcohol consumption was quantified according to a previous study.^33^ Patients self‐reported weekly (for frequent drinkers) or monthly (for infrequent drinkers) alcohol consumption in six categories (red wine, white wine/champagne, beer/cider, spirits, fortified wine, and “other”). According to UK Biobank, 1 glass of red wine, white wine, fortified wine, or “other” is equivalent to 2 units of alcohol; 1 pint of beer/cider is equivalent to 2 units of alcohol; 1 single shot of spirits is equivalent to 1 unit. Weekly/monthly consumptions were converted into units of alcohol consumed per day and summed over all categories of alcohol. If a patient did not report any alcohol consumption or a patient reported never drinking alcohol (field 1558), daily alcohol consumption was set to 0 units.

Three markers of cardiac and arterial functions were used: left ventricle (LV) ejection fraction (field 22420); maximum carotid intima‐media thickness (IMT) calculated by taking the maximum value of the maximum carotid IMT at 120, 150, 210, and 240 degrees (fields 22672, 22675, 22678, 22681, respectively); and the arterial stiffness index (ASI; field 21021).

### 2.3 Incident dementia

Incident dementia was ascertained by searching for dementia‐related International Classification of Diseases version 10 (ICD‐10) codes in the hospital records and mortality data and Read V2 codes in the primary care data.^34^ Participants whose dementia occurred before the first imaging visit were excluded. Time to dementia was duration from date of first imaging visit (field 53) to date of dementia (field 42018). For participants who were censored, that is, without recorded dementia, censoring time was defined between the first imaging visit date and October 13, 2023, when dementia data were downloaded for the analysis of this study. If a censored participant died after the first imaging visit, censoring time was defined between the first imaging visit and date of death (field 40000).

### 2.4 MRI analysis

The MRI sequence parameters are provided in Table S1 in supporting information.

We analyzed four markers of CSF dynamics. For two, DTI‐ALPS and BOLD‐CSF, we have automated the process to extract these markers in UK Biobank, and in each case validated these new analysis systems.

#### 2.4.1 PVS volume

**FIGURE 1:**
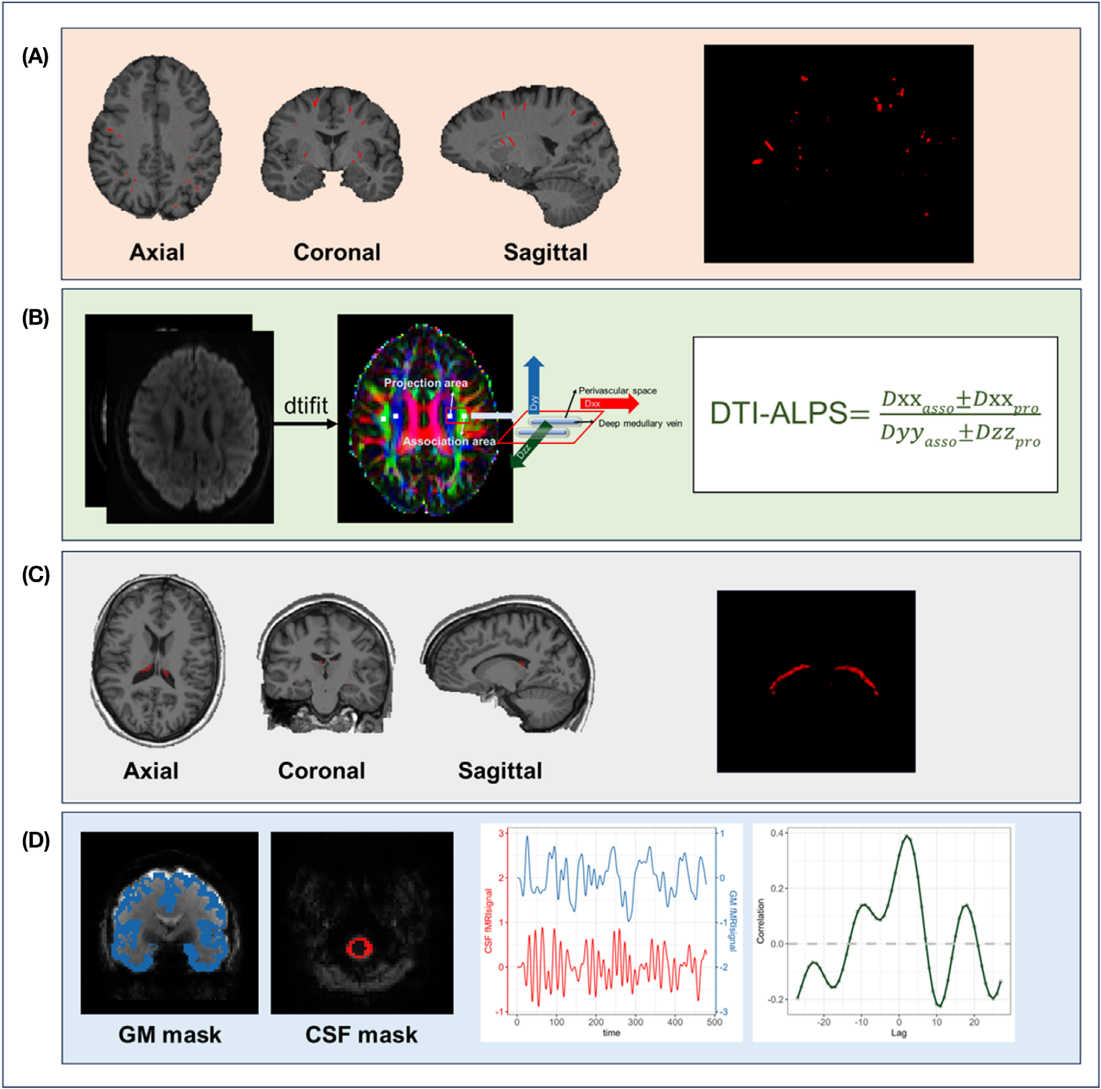
The illustration of non‐invasive MRI proxies of CSF dynamics markers. A, PVS volume automatic segmentation using deep learning. B, DTI‐ALPS calculation using the diffusivities of water molecules along the medullary veins in the centrum semiovale region, normalized by diffusivities along the projection and association fibers. C, CP volume automatic segmentation using FreeSurfer. D, BOLD‐CSF coupling evaluation by measuring the maximal cross‐correlation between CSF signals (red trace) and BOLD signals (blue trace) in the cortex. BOLD, blood oxygen level dependent; CP, choroid plexus; CSF, cerebrospinal fluid; DTI‐ALPS, diffusion tensor image analysis along the perivascular space; GM, gray matter; MRI, magnetic resonance imaging; PVS, perivascular space.

Whole brain PVS volume (Figure 1A) was calculated automatically using a pretrained deep learning model, previously validated against expert human observers.^35^, ^36^ To calculate PVS volume, bias correction and skull stripping were performed on raw T1 images.^37^ Intensity normalization was performed using a Fuzzy C‐means method. PVS volume was calculated as the sum of the number of voxels in the PVS segmentation mask multiplied by voxel dimension.

#### 2.4.2 DTI‐ALPS calculation

DTI‐ALPS (Figure 1B) was calculated using an in‐house automated analysis pipeline from dMRI data (UK Biobank field 20250). Topup correction, motion correction, and eddy correction were performed using the eddy command in FSL.^27^ Only the shell with a *b* value of 0 and 1000 s/mm^2^ was selected for further analysis. The diffusion tensor was fitted to the preprocessed DTI data using the dtifit command in FSL.^38^ Fractional anisotropy (FA) was calculated from the diffusion tensor. The FA map was registered to the FMIRB58 FA template using symmetric normalization from the ANTs package.^39^ The resulting transform was used to warp each component of the diffusion tensor to the Montreal Neurological Institute (MNI)152 template. The region of interest (ROI) used to calculate DTI‐ALPS was obtained based on a previous paper.^40^ Four ROIs were used: two on the projection fibers and two on the association fibers on both sides. All ROIs were spherical with a radius of 2.5 mm.

To prevent regions with severe white matter lesions or lacunes from affecting DTI‐ALPS calculation, these regions were excluded as follows: FA was calculated from the diffusion tensor registered to the MNI space. Atropos algorithm was used to classify each voxel in the FA map into whether it is white matter or not white matter.^41^ Voxels with < 90% probability of being white matter were excluded. To further ensure that potential lacunes and white matter lesions were avoided, each ROI was displayed between 1 to 2 voxels in each direction along the *x*–*y* plane. The displacement that yielded the highest sum of white matter probability over the ROI was chosen as the final place for the ROI. An example of ROI placement determined by this method is displayed in Figure S2 in supporting information. DTI‐ALPS was calculated as described previously.^11^ Before implementing our automatic methods for calculation of DTI‐ALPS it in the UK Biobank data we validated it by comparing it to the previously used and published manual method in four cohorts.^40^ Validation of this automatic method against the gold standard is described in Tables S2 and S3 and Figure S3 in supporting information.

#### 2.4.3 CP volume

CP volume (Figure 1C) was calculated by adding the volumes of left and right CP (UK Biobank fields 26567 and 26598).

#### 2.4.4 BOLD‐CSF coupling calculation

To calculate BOLD‐CSF coupling (Figure 1D) automatically, we preprocessed rs‐fMRI data (UK Biobank field 20225) using the method of Han et al.^21^ The slice timing correction (FSL slicetimer^38^ command) and motion correction (FSL mcflirt command) were performed on the raw rs‐fMRI volumes using FSL (Figure S4 in supporting information). The first rs‐fMRI volume was segmented by the FreeSurfer mri_synthseg command. Cortical gray matter mask was obtained using FreeSurfer labels 3 and 42. The CSF mask was automatically segmented using an in‐house developed deep learning algorithm (Figures S5 and S6, and Tables S4 and S5 in supporting information). After segmentation, the first and last five fMRI volumes were discarded to allow magnetization to reach steady states.

To obtain the global BOLD signals, Gaussian filtering was performed on all rs‐fMRI volumes with a full width at half maximum (FWHM) of 4 mm. Linear detrending was then performed using generalized linear regression. The detrended data were temporally filtered between 0.01 and 0.1 Hz. After temporal filtering, each voxel was *z* transformed. Within the mask of the cortical gray matter, voxel intensities were averaged at each time point. For each timeframe, we calculated the mean cortical intensity difference between the current timeframe and its previous timeframe, representing the first derivative of the mean cortical intensity over time.

To extract the CSF signals, linear detrending was performed on the preprocessed rs‐fMRI images before Gaussian smoothing. The detrended data were temporally filtered between 0.01 and 0.1 Hz. After temporal filtering, each voxel was *z* transformed. Within the mask of the CSF in the bottom three slices of the rs‐fMRI images for justification of using the bottom three slices, voxel intensities were averaged at each time point to obtain the CSF signal intensity changes over time.

To calculate the coupling between BOLD and CSF signals, the BOLD signal was multiplied by −1 and all values < 0 were set to 0.^12^ The BOLD signal was shifted with respect to the CSF signal with an increment of one repetition time, with the degree of shift constrained between −20 seconds and 20 seconds. BOLD‐CSF coupling was defined as the maximal correlation obtained among the different shifts, and the lag time was the amount of shift that corresponds to the maximal correlation.

Before implementing our automatic methods for calculation of BOLD‐CSF coupling in the UK Biobank data, we validated it by comparing it to the previously used and published manual method in Alzheimer's Disease Neuroimaging Initiative (ADNI) and UK Biobank cohorts. Validation of this automatic method against the gold standard was described in the Table S6 in supporting information.

#### 2.4.5 Other imaging markers

White matter hyperintensity (WMH) volume was derived from the UK Biobank field 25781.^42^ Mean diffusivity (MD) was used. FA maps were obtained using the FSL dtifit command. Free water (FW) and FW‐corrected FA (cFA) were obtained using the scripts in a previous study.^43^ cFA was segmented by the atropos command in ANTs^41^ into white matter and non‐white matter tissues. The resulting white matter probability map was used to derive a gray matter probability map by: 1 – white matter probability – free water. The white matter probability map was multiplied by 2 and added to the gray matter probability map to create a pseudo‐T1 map. This map was parcellated by FreeSurfer. Median values for MD were calculated in the entire cerebral white matter regions (FreeSurfer labels 2 and 41). Brain atrophy was defined as the parenchymal volume fraction, which is the sum of gray matter (UK Biobank field 25006) and white matter volumes (field 25008) divided by total intracranial volume (field 26521).

### 2.5 Statistical analysis

Statistical analyses were performed in R (version 4.1.2). For demographics, cardiovascular risk factors, WMH, and MRI imaging markers, Shapiro–Wilk test was performed to assess normality for continuous variables. If the variable is normally distributed, mean and standard deviation were used. For variables not normally distributed, median and interquartile range were used. WMH was normalized and log‐transformed. CP volume was normalized by total brain volume.

Correlation analysis was performed to analyze associations of each MRI‐based proxy of CSF dynamics with each other and with demographics, parenchymal volume fraction, and risk factors. Pearson correlation was used if the variable is normally distributed, otherwise Spearman correlation was applied. If a correlation was significant (*p* < 0.05), linear regression was performed adjusting for age and sex. For each category of variables (neuroimaging markers, cardiovascular risk factors, cardiac function markers), multiple testing correction was performed using the Benjamini–Hochberg method.

To investigate whether CSF dynamics imaging markers predicted incident dementia, a proportional subdistribution hazards model was used. Events of death were incorporated as a competing risk. All analyses were adjusted for age, sex, and education. The R package “cmprsk” was used. We additionally included two sensitivity analyses: Model 1 (adjusting for age, sex, education, and median white matter MD), Model 2 (adjusting for age, sex, education, median white matter MD, hypertension status, diabetes status, pack‐years of smoking, daily alcohol consumption, insomnia status, and apolipoprotein E [*APOE*] ε4 carrier status). *APOE* genotype was determined by single‐nucleotide polymorphism data for rs429358 and rs7412. Insomnia status was obtained from field 1200. Multiple testing correction was performed using the Benjamini–Hochberg method.

A further sensitivity analysis was performed after excluding participants who developed dementia within 1 year of their MRI visits, with adjustments of age, sex, and years of education.

To investigate whether different risk factors can predict dementia conversion, a proportional subdistribution hazards model was used adjusting for age, sex, and education. The proportional hazard assumption was evaluated using the Schoenfeld residual test. Multiple testing correction was performed using the Benjamini–Hochberg method.

To investigate whether CSF dynamics imaging markers mediated the relationship between risk factors and dementia conversion, causal mediation analysis (CMA) was performed using the “CMAverse” package in R. Each mediation analysis was adjusted for age, sex, and education. Missing data in either the mediator, dependent, or independent variables were discarded. Direct effect, indirect effect, total effect, and proportion of indirect effect in total effect were computed. Multiple testing correction was performed using the Benjamini–Hochberg method.

## 3 RESULTS

### 3.1 Demographics

Forty‐four thousand three hundred eighty‐four participants with MRI data were included, of whom 41,477 had T1 images enabling CP volume quantification, 39,173 had DTI images passing quality control for DTI‐ALPS calculation, and 37,906 had rs‐fMRI passing quality control for BOLD‐CSF coupling calculation (Figure S1). Demographics are shown in Table 1, and demographics for the subset of participants with available T1, dMRI, or rs‐fMRI data are shown in Table S7 in supporting information.

**TABLE 1:** Characteristics of participants.

**Demographics**	
Age (years), median (IQR)	65.0 (58.0–70.0)
Male, n (%)	21253 (47.9)
Education (years), median (IQR)	19.0 (10.0–20.0)
Follow‐up time for dementia (years), median (IQR)	5.3 (4.5–6.8)
**Cardiovascular risk factors**	
Systolic blood pressure (mmHg), median (IQR)	140.0 (127.0–154.0)
Diastolic blood pressure (mmHg), median (IQR)	78.0 (72.0–86.0)
Pulse pressure (mmHg), median (IQR)	60.0 (51.0–72.0)
Diabetes status, n (%)	2173 (4.9)
Smoking (pack‐years), median (IQR)	0.0 (0.0–0.0)
Alcohol (units/day), median (IQR)	1.4 (0.3–2.9)
**Cardiac and arterial function markers**	
LV ejection fraction (%), median (IQR)	56.0 (52.0–60.0)
Maximum carotid IMT (µm), median (IQR)	885.0 (770.0–1039.0)
ASI, median (IQR)	9.4 (7.4–11.4)
**Imaging markers**	
WMH volume (mL), median (IQR)	2.9 (1.5–5.8)
PVS volume (mL), median (IQR)	2.0 (1.3–3.1)
DTI‐ALPS, median (IQR)	1.5 (1.4–1.6)
CP volume (mL), median (IQR)	1.3 (1.0–1.7)
BOLD‐CSF coupling, median (IQR)	0.5 (0.3–0.6)
**Dementia conversion**	
Dementia, n (%)	133 (0.3)

*Note*: Data were presented as median (interquartile range) or number (percentage, %).

Abbreviations: ASI, arterial stiffness index; BOLD, blood oxygen level dependent; CP, choroid plexus; CSF, cerebrospinal fluid; DTI‐ALPS, diffusion tensor image analysis along the perivascular space; IMT, intima‐media thickness; IQR, interquartile range; LV, left ventricle; PVS, perivascular space; WMH, white matter hyperintensity.

### 3.2 Validation of automated MRI analysis techniques

We demonstrated high correlations between manual and automated methods for measuring DT1‐ALPS (correlation coefficient 0.85–0.89, Table S3) and BOLD‐CSF coupling (correlation coefficient 0.86–0.94, Table S6).

### 3.3 Correlation between four MRI‐based proxies of CSF dynamics

We found significant correlations between four MRI‐based proxies of CSF dynamics, except that BOLD‐CSF coupling and PVS volume were not correlated (Table S8 in supporting information).

### 3.4 Associations between MRI‐based proxies of CSF dynamics and risk factors

#### 3.4.1 Association of MRI‐based proxies of CSF dynamics with demographics and brain atrophy

**FIGURE 2:**
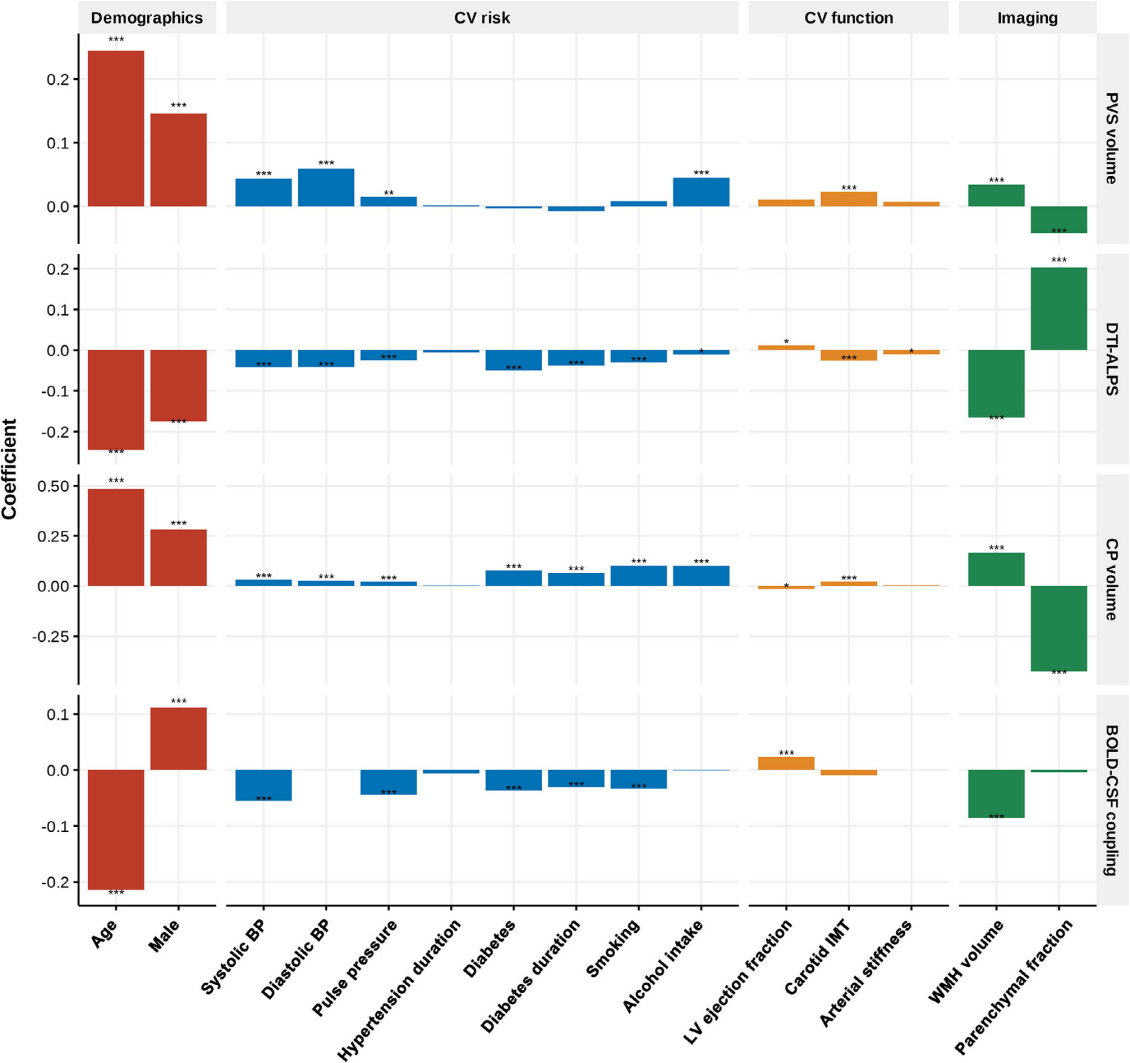
The association of MRI proxies of CSF dynamics with demographics and risk factors. BOLD, blood oxygen level dependent; BP, blood pressure; CP, choroid plexus; CSF, cerebrospinal fluid; CV, cardiovascular; DTI‐ALPS, diffusion tensor image analysis along the perivascular space; IMT, intima‐media thickness; LV, left ventricle; MRI, magnetic resonance imaging; PVS, perivascular space; WMH, white matter hyperintensity.

Higher age was associated with impaired CSF dynamics as measured by lower DTI‐ALPS (β = −0.245, *p* < 0.001), lower BOLD‐CSF coupling (*β* = −0.214, *p* < 0.001), higher normalized CP volume (*β* = 0.484, *p* < 0.001), and higher PVS volume (*β* = 0.245, *p* < 0.001). Males showed lower DTI‐ALPS (*β* = −0.175, *p* < 0.001), higher CP volume (*β* = 0.281, *p* < 0.001), and higher PVS volume (*β* = 0.146, *p* < 0.001), and females showed lower BOLD‐CSF coupling (*β* = 0.111, *p* < 0.001). Higher parenchymal volume fraction was significantly associated with lower PVS volume (*β *= −0.042, *p* < 0.001), higher DTI‐ALPS (*β* = 0.203, *p* < 0.001), and lower CP volume (*β* = −0.424, *p* < 0.001), but not BOLD‐CSF coupling (*β* = −0.004, *p* = 0.427; Figure 2).

#### 3.4.2 Association of cardiovascular risk factors with MRI‐based proxies of CSF dynamics

Many cardiovascular risk factors were associated with markers of impaired CSF dynamics. Associations were consistent with BP. Higher systolic BP was associated with higher PVS volume (*β* = 0.044, *p* < 0.001), lower DTI‐ALPS (*β* = −0.042, *p* < 0.001), higher CP volume (*β* = 0.032, *p* < 0.001), and lower BOLD‐CSF coupling (*β* = −0.055, *p* < 0.001). Higher pulse pressure was associated with all four MRI markers of CSF dynamics (PVS volume: *β* = 0.015, *p* = 0.006; DTI‐ALPS: *β* = −0.024, *p* < 0.001; CP volume: *β* = 0.022, *p* < 0.001; BOLD‐CSF coupling: *β* = −0.044, *p* < 0.001). Higher diastolic BP was associated with higher PVS volume (*β* = 0.059, *p* < 0.001), lower DTI‐ALPS (*β* = −0.041, *p* < 0.001) and higher CP volume (*β* = 0.027, *p* < 0.001), but no association was observed between diastolic BP and BOLD‐CSF coupling. The duration of exposure to hypertension was not associated with MRI‐based proxies of CSF dynamics (Figure 2).

Diabetes was associated with lower DTI‐ALPS (*β* = −0.049, *p* < 0.001), higher CP volume (*β* = −0.077, *p* < 0.001), and lower BOLD‐CSF coupling (*β* = −0.037, *p* < 0.001), but not PVS volume (*β* = −0.003, *p* = 0.542). Similarly, the duration of exposure to diabetes was associated with lower DTI‐ALPS (*β* = −0.038, *p* < 0.001), higher CP volume (*β* = 0.065, *p* < 0.001), and lower BOLD‐CSF coupling (*β* = −0.030, *p* < 0.001) but not PVS volume (*β* = −0.008, *p* = 0.154). Smoking was associated with lower DTI‐ALPS (*β* = −0.030, *p* < 0.001), higher CP volume (*β* = 0.101, *p* < 0.001), and lower BOLD‐CSF coupling (*β* = −0.034, *p* < 0.001). Alcohol was associated with higher PVS volume (*β* = 0.045, *p* < 0.001), lower DTI‐ALPS (*β* = −0.010, *p* = 0.036), and higher CP volume (*β* = 0.100, *p* < 0.001; Figure 2).

#### 3.4.3 Association of MRI‐based proxies of CSF dynamics with markers of cardiac and arterial injury and WMH

Higher maximum carotid IMT was associated with higher PVS volume (*β* = 0.021, *p* < 0.001), lower DTI‐ALPS (*β* = −0.026, *p* < 0.001), higher CP volume (*β* = 0.022, *p* < 0.001), but not BOLD‐CSF coupling (*β* = −0.009, *p* = 0.094). Lower LV ejection fraction was associated with lower DTI‐ALPS (*β* = 0.011, *p* = 0.046), higher CP volume (*β* = −0.015, *p* = 0.017), and lower BOLD‐CSF coupling (*β* = 0.023, *p* < 0.001). Increased arterial stiffness was associated with both lower DTI‐ALPS (*β* = −0.010, *p* = 0.033), but not with BOLD‐CSF coupling, PVS volume, or CP volume (Figure 2). Higher WMH volume was associated with higher PVS volume (*β* = 0.034, *p* < 0.001), lower DTI‐ALPS (*β* = −0.166, *p* < 0.001), higher CP volume (*β* = 0.165, *p* < 0.001), and lower BOLD‐CSF coupling (*β* = −0.086, *p* < 0.001; Figure 2). Additionally, we found that WMH volume was significantly associated with cardiac and/or vascular function markers (LV ejection fraction: *β* = −0.033, *p* < 0.001; arterial stiffness: *β* = 0.016, *p* < 0.001; carotid IMT: *β* = 0.048, *p* < 0.001), and MRI‐based proxy of CSF dynamics mediated this association (Figure S7 in supporting information).

### 3.5 Do MRI‐based proxies of CSF dynamics markers predict dementia conversion?

During the median 5.3 years of follow‐up, 133 cases of incident dementia occurred. Higher DTI‐ALPS at baseline predicted lower risk of dementia conversion after correcting for age, sex, and education (hazard ratio [HR] = 0.866 [0.797–0.942], *p* = 0.001; Table 2). Higher CP volume predicted dementia conversion after correcting for age, sex, and education (HR = 1.185 [1.088–1.291], *p* < 0.001). Lower BOLD‐CSF coupling predicted dementia conversion (HR = 0.875 [0.806–0.951], *p* = 0.001). These results remain significant after adjusting for white matter MD, cardiovascular risk factors, and *APOE* ε4 carrier status (Table S9 in supporting information). PVS volume did not predict dementia conversion (HR = 1.013 [0.940–1.091], *p* = 0.730; Table 2). To account for the latency period of dementia development, an additional sensitivity analysis after excluding participants who developed dementia within 1 year of MRI visits showed similar results (Table S10 in supporting information).

**TABLE 2:** MRI‐based proxies of CSF dynamics in predicting dementia.

Markers	HR (95% CI)	*p* value	Schonfield residual test *p* value
PVS volume	1.013 (0.940–1.091)	0.730	0.536
DTI‐ALPS	0.866 (0.797–0.942)	0.001^*^	0.536
CP volume	1.185 (1.088–1.291)	<0.001^*^	0.913
BOLD‐CSF coupling	0.875 (0.806–0.951)	0.001^*^	0.536

*Note*: All analysis was corrected for age, sex, and education. Schonfield residual test *p* value > 0.05 suggests the validity of proportional hazard model.

Abbreviations: BOLD, blood oxygen level dependent; CI, confidence interval; CP, choroid plexus; CSF, cerebrospinal fluid; DTI‐ALPS, diffusion tensor image analysis along the perivascular space; HR, hazard ratio; MRI, magnetic resonance imaging; PVS, perivascular space.

* *P* < 0.05 after Benjamini–Hochberg correction.

### 3.6 Prediction of incident dementia by cardiovascular risk factors and markers of cardiovascular damage

Diabetes (HR = 1.118 [1.060–1.178], *p* < 0.001), duration of diabetes (HR = 1.060 [1.024–1.097], *p* = 0.002), duration of hypertension (HR = 1.142 [1.089–1.199], *p* < 0.001), pack‐years of smoking (HR = 1.212 [1.158–1.268], *p* < 0.001), LV ejection fraction (HR = 0.861 [0.807–0.918], *p* < 0.001), and WMH volume (HR = 1.117 [1.059–1.179], *p* < 0.001) predicted dementia conversion (Table S11 in supporting information). Additional sensitivity analysis after excluding participants who developed dementia within 1 year of MRI visits showed similar results (Table S10).

### 3.7 Do MRI‐based proxies of CSF dynamics imaging markers mediate the effect of risk factors on predicting dementia?

**FIGURE 3:**
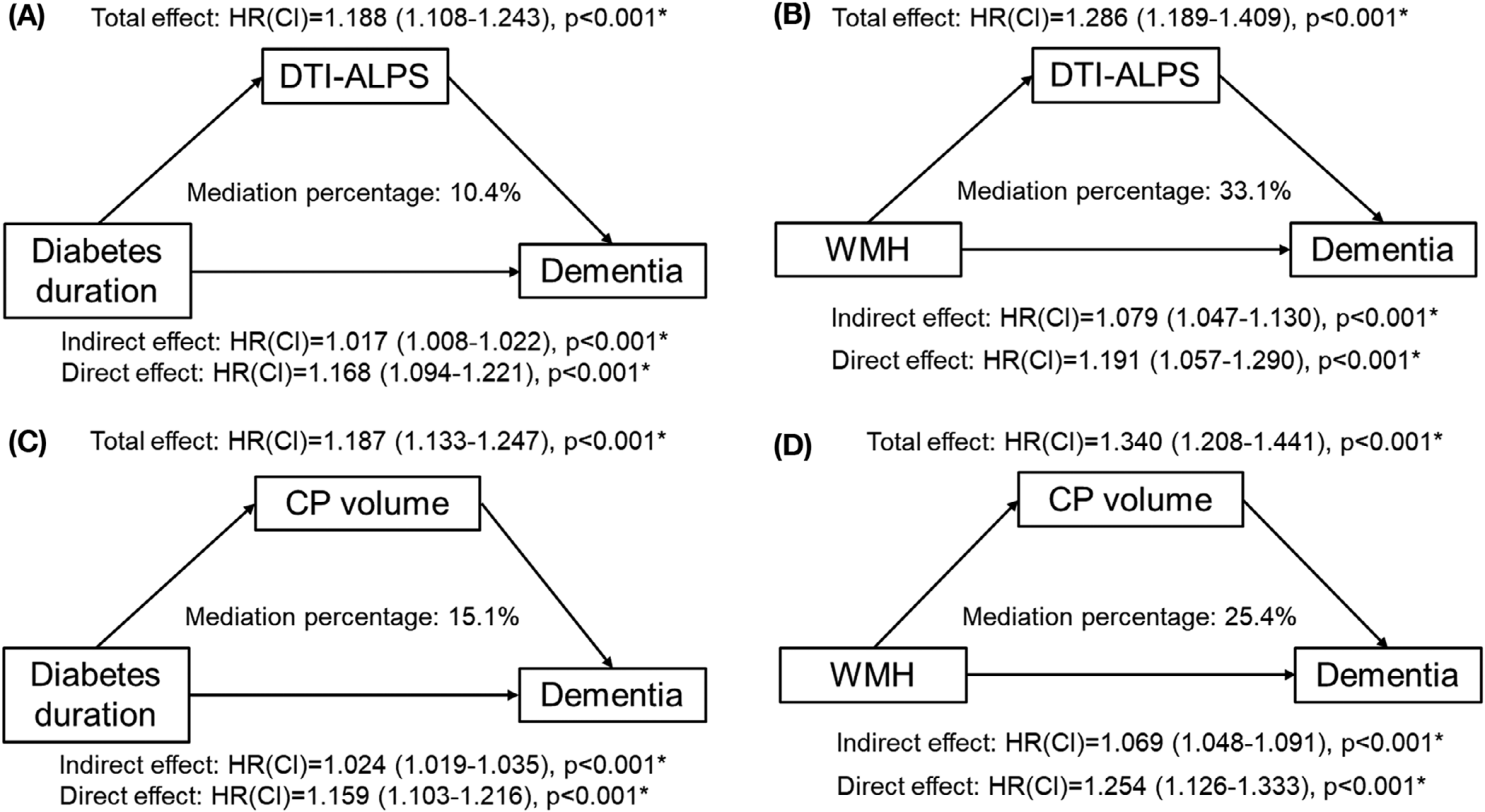
MRI proxies of CSF dynamics mediate the association between cardiovascular risk factors and dementia. A, DTI‐ALPS mediates the association between diabetes duration and dementia. B, DTI‐ALPS mediates the association between WMH and dementia. C, CP volume mediates the association between diabetes duration and dementia. D, CP volume mediates the association between WMH and dementia. CI, confidence interval; CP, choroid plexus; CSF, cerebrospinal fluid; DTI‐ALPS, diffusion tensor image analysis along the perivascular space; HR, hazard ratio; MRI, magnetic resonance imaging; WMH, white matter hyperintensities.

Both DTI‐ALPS and CP volume partially mediated the association between WMH volume and dementia (DTI‐ALPS: indirect effect = 1.079 [1.047–1.130], *p* < 0.001, mediation proportion = 33.1%; CP volume: indirect effect = 1.069 [1.048–1.091], *p* < 0.001, mediation proportion = 25.40%; Figure 3). The mediation for DTI‐ALPS remained after additional correction for median white matter MD (indirect effect = 1.064 [1.023–1.117], *p* < 0.001, mediation proportion = 34.6%; Table S12 in supporting information). Both DTI‐ALPS and CP volume partially mediated the association between diabetes duration and dementia (DTI‐ALPS: indirect effect = 1.017 [1.008–1.022], *p* < 0.001, mediation proportion 10.4%; CP volume: indirect effect = 1.024 [1.019–1.035], *p* < 0.001, mediation proportion 15.1%; Figure 3).

## 4 DISCUSSION

To investigate whether MRI‐based proxies of CSF dynamics predict dementia risk, we have automated the process of quantifying DTI‐ALPS and BOLD‐CSF coupling in large datasets. MRI‐based proxies of CSF dynamics were intercorrelated, except for BOLD‐CSF coupling and PVS volume. Impaired CSF dynamics measured by DTI‐ALPS, CP volume, and BOLD‐CSF coupling, but not by PVS volume, predicted incident all‐cause dementia. All imaging markers were associated with cardiovascular risk factors, cardiac and arterial function markers, and WMHs, although the strength of these associations varied. In mediation analysis, CP volume and DTI‐ALPS partially mediated the associations between WMHs and diabetes duration with dementia.

PVS volume, DTI‐ALPS, BOLD‐CSF coupling, and CP volume are recently proposed non‐invasive imaging markers, each providing insights into different aspects of CSF dynamics. Among them, BOLD‐CSF coupling is from the assumption that fluctuations in cerebral hemodynamics drive CSF flow. The initial study using this method reported widespread, high‐amplitude spontaneous brain activations during drowsiness and sleep, reflected as large global signal peaks in rs‐fMRI, which were coupled with CSF movement.^12^ Another study demonstrated CSF movement is facilitated by changes in cerebral blood volume, primarily within the low‐frequency range, even when individuals are awake.^44^ These findings support the use of this method for assessing CSF dynamics in both sleep and wakeful states. Our observed associations between BOLD‐CSF coupling and dementia are consistent with previous studies that identified correlations with cognition,^20^, ^21^ supporting a role of CSF disruption in dementia development. However, BOLD‐CSF coupling reflects an overall alteration in CSF flow and does not pinpoint which specific component of CSF dynamics is impaired.

DTI‐ALPS, derived from dMRI, evaluates diffusivity parallel to PVS surrounding medullary veins at the level of the lateral ventricles.^11^ It correlates well with the clearance component of CSF dynamics—specifically glymphatic clearance—as validated by intrathecal injection studies.^45^ Previous research in AD^46^ and cerebral small vessel disease (CSVD)^17^ demonstrated DTI‐ALPS is a predictor of dementia. Taken together with our findings, this supports the hypothesis that impaired CSF dynamics along venous pathways—also referred to as glymphatic clearance dysfunction—plays a role in dementia. To address methodological confounds, we adjusted for MD, another diffusion metric linked to dementia, and the association between DTI‐ALPS and dementia remained significant, indicating DTI‐ALPS reflects more than general white matter microstructure damage.

The CP not only produces CSF but also forms the blood–CSF barrier supporting nutrients, waste clearance, and immune surveillance.^47^ Structural changes in the CP have been reported in autopsy studies of elderly individuals and AD.^48^ An enlarged CP was associated with reduced CSF production and decreased capacity to clear metabolic waste.^14^ We observed CP volume increases with age, cardiovascular risk factors, and markers of cardiovascular damage, and that larger CP volume predicts increased risk of future dementia. This association may reflect a compromised ability of the CP to drive CSF dynamics or support the clearance of waste products from the brain via the CSF.

Enlarged PVS are observed parallel to small arterioles,^49^, ^50^ appear hypointense on T1WI and hyperintense on T2‐weighted images (T2WI), and reflect fluid stagnation within the spaces, suggesting CSF inflow dysfunction.^10^ They are increased in dementia particularly due to CSVD.^51^ Previous studies have reported both positive and negative associations between PVS and cognitive impairment or dementia.^17^, ^52^, ^53^ Our findings demonstrated no association between PVS burden and dementia, consistent with two prior meta‐analyses.^54^, ^55^ This suggests that, compared to DTI‐ALPS and CP volume—which could reflect CSF clearance function—PVS volume, representing CSF inflow capacity,^49^ may play a more limited role in the pathogenesis of dementia.^52^

Different MRI‐based proxies of CSF dynamics were correlated with each other, reflecting that they measure different aspects of the same CSF circulation system. The lower correlation of BOLD‐CSF coupling with other markers may be because BOLD‐CSF measures fluctuations of CSF inflow outside the brain parenchyma, whereas the other markers reflect CSF dynamics closer to the brain parenchyma.

We found associations between markers of cardiac and arterial function with impaired CSF dynamics. Animal studies have demonstrated cerebral hemodynamics are closely linked to CSF dynamics.^56^, ^57^ It has been hypothesized that cardiac and arterial function play a crucial role in CSF flow via arterial pulsation.^56^ Arteries exhibit rhythmic pulsations that synchronize with the heartbeat and propagate as waves along arteries.^58^ Both cardiac dysfunction and arterial stiffness could impact arterial pulsation, diminishing the driving force for CSF flow.^10^ However, cardiac and arterial function markers exhibited the lowest effect sizes among all cardiovascular‐related variables. While cardiac output and large artery compliance contribute to the overall pulsatile flow of blood to the brain, they are not the primary drivers of CSF dynamics within the brain parenchyma. Pulsatility transmitted through cerebral small vessels is thought to play a more direct role in regulating CSF movement and interstitial fluid exchange.^59^ At present, methods for directly assessing cerebral small vessel function are limited and often feasible only in small‐scale studies.^60^

We found significant associations between cardiovascular risk factors and MRI proxies of CSF dynamics. Among these, BP exhibited the largest effect size on PVS volume. This may be explained by the fact that arteriolosclerosis—a common consequence of hypertension^61^—directly contributes to PVS enlargement. In contrast, diabetes, smoking, and alcohol showed higher effect sizes on CP volume than hypertension. While hypertension and diabetes are known to affect CP structure,^62^, ^63^ evidence linking smoking or alcohol to CP volume is limited. One hypothesis is that ethanol and nicotine‐related byproducts may influence CP structure and function, possibly through inflammatory pathways.^64^, ^65^

Among cardiovascular damage markers, WMH had the greatest effect on CSF dynamics. Furthermore, the association between WMH and dementia was significantly mediated by both DTI‐ALPS and CP volume. WMHs are predictors of dementia in population‐based studies,^24^ and reflect CSVD.^66^ Our data suggest WMHs represent the end point of cerebrovascular injury, perhaps diminishing the driving force of CSF circulation from small vessels and contributing to cognitive decline.^67^ As WMHs represent in‐brain vascular dysfunction, this may explain why WMHs are associated most strongly with impaired CSF dynamics.

Both DTI‐ALPS and CP volume mediated the association between diabetes duration and dementia risk, suggesting effective management of diabetes may delay dementia progression by improving CSF dynamics. Evidence from diabetes animal models has shown transporter expression changes in CP, which affect CSF production.^63^ The glucagon‐like peptide‐1 (GLP1) receptor agonists, used in obesity and diabetes, reduced intracranial pressure in patients with idiopathic intracranial hypertension via CP CSF production.^68^ Trials are examining GLP1 agonists in AD.

Our study has a number of strengths. We developed automated analysis methods to measure DTI‐ALPS and BOLD‐CSF coupling in large datasets, allowing us to determine imaging markers in 40,000 individuals, a much larger sample than previous studies. We examined associations with incident dementia over a follow‐up period of > 5 years, increasing the likelihood that the observed associations reflect potential causal relationships. Additional sensitivity analyses controlling for vascular risk factors and *APOE* ε4 support the robustness of these imaging markers in predicting dementia.

However, our study also has limitations. Dementia encompasses a spectrum of neurodegenerative diseases, and impairments in CSF dynamics may vary across different dementia subtypes.^69^ Our ability to analyze associations by subtypes was limited by the small number of cases for specific dementia subtypes in our cohort. Among 44,385 participants, there were only 24 confirmed cases of vascular dementia and 4 of frontotemporal dementia. Additionally, dementia diagnoses were derived from hospital and general practitioner records, which may reduce diagnostic accuracy. Second, we did not assess how well the risk factors were controlled and whether this could influence the proxies of CSF dynamics. Future studies could use prescription databases to determine whether specific anti‐hypertensive or anti‐diabetic medications are associated with improved CSF dynamics. Third, the moderate follow‐up duration may limit the ability to detect associations with slowly developing conditions such as dementia. Last, the proxies of CSF dynamics markers used are indirect and each has methodological limitations. DTI‐ALPS can be influenced by white matter microstructure.^70^ We addressed this concern by adjusting for whole‐brain microstructure using MD as a covariate. Furthermore, DTI‐ALPS uses ROI placement, thereby capturing only regional, rather than whole‐brain, CSF dynamics.^71^ For CP volume, we used data from UK Biobank, in which segmentation was performed using FreeSurfer on T1WI. This single‐modality approach may limit segmentation accuracy, as CP delineation can be improved with multi‐contrast imaging.^72^ BOLD‐CSF coupling is derived from rs‐ fMRI, which is limited by a low signal‐to‐noise ratio. Although our analysis focused on the low‐frequency range—primarily driven by physiological processes such as neuronal activity—non‐neuronal fluctuations have been reported to influence BOLD signals, potentially affecting the reliability of this metric.^73^, ^74^ Last, PVS segmentation, incorporating both T1WI and T2WI, improves segmentation accuracy.^75^ However, UK Biobank did not collect T2WI.

In conclusion, we demonstrated three MRI proxies of CSF dynamics markers, BOLD‐CSF coupling, DTI‐ALPS, and CP volume, predict future dementia risk. Cardiovascular risk factors were predictors of impaired CSF dynamics, and the association between CSF dynamics and dementia was mediated by WMH and diabetes. Strategies to improve CSF dynamics may reduce dementia risk, although this needs testing in intervention studies.

## CONFLICT OF INTEREST STATEMENT

All authors declare no conflicts of interest. Author disclosures are available in the supporting information.

## CONSENT STATEMENT

All procedures performed in studies involving human participants were under the ethical standards of the Institutional and National Research Committee and with the 1964 Declaration of Helsinki and its later amendments or comparable ethical standards. Written informed consent was from all participants and authorized representatives. The UK Biobank is approved from the North West Multi‐centre Research Ethics Committee as a Research Tissue Bank and researchers do not require separate ethical clearance. The current study was conducted under the approved application number 36509. Approval for the MINERVA trial was granted by the East of England, Cambridge Central Research Ethics Committee (REC no: 18/EE/0237). The MRC‐PET cohort was approved by the East of England—Cambridge South Ethics Committee (REC no: 16/EE/0468), and the Administration of Radioactive Substances Advisory Committee (ARSAC ref: 83/3886/35752). The SCANS cohort was registered (REC no: 07/Q0803/82) and approved by a local research ethics committee (LondonWandsworth). ADNI was approved by the institutional review boards of all participating institutions. All ADNI participants provided written informed consent according to the Declaration of Helsinki before study enrolment.

## Supporting information



Supporting information

Supporting information
