# Supplementary material for: MRI markers of cerebrospinal fluid dynamics predict dementia and mediate the impact of cardiovascular risk
Source: Alzheimers Dement. 2025 Oct 23;21(10):e70699. doi: 10.1002/alz.70699 (PMC12547625; doi:10.1002/alz.70699)
Supplement: Supplementary file 2 — Supporting information [file ALZ-21-e70699-s001.docx]

# Supplementary Materials

**Supplementary Methods** (page 4-5)

**Supplementary Results** (page 6)

**Table S1** MRI sequence parameters. (page 7)

**Table S2** Demographics of the participants in the datasets used to validate the automatic calculation of DTI-ALPS. (page 8)

**Table S3** Correlation between manual and automatic calculations of DTI-ALPS, and correlation between the error of automatic method and normalised WMH volume. (page 9)

**Table S4** Demographics of the participants in the datasets used to validate the automatic calculation of BOLD-CSF coupling. (page 10)

**Table S5** Mean dice score of deep learning-segmented CSF compared with the ground truth CSF segmentation at each of the bottom slices in fMRI. (page 11)

**Table S6** Correlation between manual and automatic calculations of BOLD-CSF coupling using different numbers of bottom slices. (page 12)

**Table S7** Demographics of the participants in the UK biobank grouped by the availability of each MRI modality. (page 13)

**Table S8** Correlation between different MRI-based proxies of CSF dynamics. (page 14)

**Table S9** Sensitivity analysis for MRI-based proxies of CSF dynamics in predicting dementia. (page 15)

**Table S10** The association between risk factors and dementia after removing participants whose dementia developed within one year of MRI visit. (page 16)

**Table** **S11** The association between risk factors and dementia (page 17)

**Table S12** The mediation effect of DTI-ALPS between WMH and dementia after correcting for median WM MD (page 18)

**Figure S1** Cohort selection. (page 19)

**Figure S2** Examples of ROI placement in DTI-ALPS calculation in UKB cohort. (page 20)

**Figure S3** Correlation between manual and automatic calculations of DTI-ALPS in different datasets. (page 21)

**Figure S4** BOLD -CSF coupling algorithm. (page 22)

**Figure S5** One example of CSF segmentation (red region) using FreeSurfer on fMRI. (page 23)

**Figure S6** Examples of CSF segmentation from ADNI and UKB. f0 image: the first volume in fMRI. (page 24)

**Figure S7** MRI-based proxies of CSF dynamics mediate the association between cardiovascular functions and WMH. (page 25)

# Supplementary Methods

## Validation of DTI-ALPS calculation

1.MINocyclinE to Reduce inflammation and blood brain barrier leakage in small Vessel diseAse (MINERVA): participants with symptomatic cerebral small vessel disease (cSVD): clinical syndrome compatible with cSVD (lacunar stroke, cognitive impairment or gait apraxia) and at least moderately severe WMHs (Fazekas grade ⩾2).[1]

2.MRC-PET: three groups of participants were recruited[2]:

a.Normal control: No history of stroke or other major neurological disorder

b. Sporadic cSVD: lacunar stroke syndrome with an anatomically corresponding lacunar infarct (≤1.5 cm diameter) on MRI, either on a DWI image for scans within 3 weeks of stroke, or a cavity on T1/FLAIR for scans after 3 weeks post stroke. In addition participants had to have confluent white matter hyperintensities (WMH) defined as Fazekas grade ≥2.[3]

c.Cerebral Autosomal Dominant Arteriopathy with Subcortical Infarcts and Leukoencephalopathy (CADASIL): confirmed genetic diagnosis of CADASIL, as defined by a typical cysteine changing NOTCH3 mutation, and age >18 years old.

3. St Georges Cognition and Neuroimaging in Stroke (SCANS): 120 participants with severe symptomatic SVD defined as a symptomatic lacunar infarct with confluent WMH (Fazekas grade ≥2).[4]

4.UK biobank: among the 42,565 participants, 20 participants were randomly selected among 1000 participants with lowest WMH volumes. Another 20 participants were selected among 5000 participants with highest WMH volumes.

As the MINERVA and MRC-PET cohorts share the same imaging parameters and cognitive testings, these two cohorts were combined and referred to as “MINERVA” in the rest of the paper. 93 participants were selected, of whom 20 were healthy controls, 20 had CADASIL and 53 had sporadic cSVD.

The demographics of the 3 cohorts used to validate the automatic DTI-ALPS calculation was shown in **Table S2**.

In each participant, DTI-ALPS was calculated using the automatic method proposed in this paper, and using the previously established methods with manual placement of 4 regions of interest in the centrum semiovale region.[5] The accuracy of the automatic method was evaluated using Pearson correlation between the automatic and manual methods. P values were calculated using Pearson correlation test.

To evaluate whether the error of the automatic method in calculating DTI-ALPS was influenced by the extent of cSVD, WMH was segmented from the FLAIR images in the Montreal Neurological Institute (MNI) space.[6] T1 images were segmented using FreeSurfer.[7] Total brain volume (TBV) was calculated by adding the gray matter (FreeSurfer labels 8, 10, 11, 12, 13, 17, 18, 26, 28, 47 49, 50, 51, 52, 53, 54, 58, and 60) and white matter volumes (FreeSurfer labels 2, 7, 16, 41 and 46). Normalized WMH volume was defined as the log transform of WMH volume divided by TBV. The error of the automatic method was defined as the absolute difference in DTI-ALPS between the automatic and manual method. Correlation between the error and normalized WMH volume was calculated using Pearson correlation. (**Supplementary Results,** **Table S3, Figure S3**)

## Automatic segmentation of brainstem CSF

The FreeSurfer synthseg command was trained to segment brain regions[7] and can fail to segment the low medullary and high cervical regions (**Figure S5**). Low medullary regions are typically present in the bottom slices of rs-fMRI images, where the CSF inflow effect is strongest.[8] In order to preserve the CSF inflow effect, accurate segmentation of the CSF in the low medullary region is needed.

A deep learning model was developed for this segmentation task. To train the deep learning model, the derivation cohort consisted of a randomly selected set of 94 participants from Alzheimer’s disease research initiative (ADNI), who underwent high resolution rs-fMRI scanning.[9] The validation cohort was 100 participants from UKB. In both cohorts, the rs-fMRI had a high spatial (≤2.5mm) and temporal resolution (<1s) **(Table S1**). Demographics of the participants included were detailed in **Table S4**. For each fMRI scan, the first volume was extracted. FreeSurfer synthseg was used to parcellate the first volume (f0 image). In the parcellation atlas, the CSF regions in the bottom 32 slices were manually corrected by a radiologist to create the ground truth parcellation atlas.

The deep learning model was the same as the one used in the FreeSurfer synthseg command.[7] The bottom 32 slices of each parcellation atlas were selected and the same method of creating training samples was used to synthesise MRI images from the parcellation atlases in the derivation cohort. Unlike Billot et al.,[7] in this study, the deep learning model was trained to classify the brain into 3 labels: CSF, brain parenchyma and brain ventricles. Otherwise, all model training parameters, including the learning rate, batch number, epoch number and optimiser settings, were the same as the original study. Examples of CSF segmentation were shown in **Figure S6**.

In both the derivation and validation cohorts, the dice score of the CSF region between the ground truth mask and the deep learning-segmented mask was calculated. Dice score was defined as two times the intersection area of two masks divided by the sum of the areas of the two masks. (**Supplementary Results, Table S5)**. After segmentation, the first and last 5 rs-fMRI volumes were discarded to allow magnetization to reach steady states.

## Validation of BOLD-CSF coupling calculation

Consistent with the development and validation of the CSF segmentation algorithm, we used the same derivation and validation cohorts–ADNI and UKB respectively–to evaluate the BOLD-CSF coupling derived from the ground truth versus deep learning-based CSF segmentations. Pearson correlation was calculated between the BOLD-CSF coupling derived from either approach. **(Supplementary Results, Table S6)**

# Supplementary Results

## DTI-ALPS calculation

In each cohort, DTI-ALPS values derived from automatic and manual methods were highly correlated with each other (r=0.85, 0.86 and 0.89 in MINERVA, SCANS and UKB respectively) (**Table S3, Figure S2**). We found no correlation between the error of DTI-ALPS with the normalised WMH volume.

## Automatic segmentation of brainstem CSF

In both cohorts, the dice score of CSF segmentation decreased towards higher slices (**Table S 5**).

## Validation of BOLD-CSF coupling calculation

In the ADNI cohort, the correlation between ground truth and automatic BOLD-CSF coupling values was highest when using the bottom 2 slices to obtain CSF signals (r=0.909), while for UKB, the correlation is highest when using the bottom 1 slice (r=0.944) (**Table S6**). In both cohorts, the correlation decreases when using more slices to obtain CSF signals, possibly because the accuracy of CSF segmentation decreased towards higher slices (**Table S5**). However, using fewer slices means that fewer CSF-containing voxels were available for averaging to obtain the CSF signals (**Table S6**). To balance between increasing the accuracy of BOLD-CSF coupling calculation, and reducing the need to remove participants who had too few CSF-containing voxels at the bottom slices, we chose to use the bottom 3 slices to obtain the CSF signals.

Across the entire UKB dataset, the BOLD-CSF coupling delay was -1.470 (IQR -2.205 to -0.735).

# Supplementary Tables

## TABLE S1 MRI sequence parameters.

| **Sequence** | **Parameter** | **MINERVA** | **SCANS** | **UKB** | **ADNI** |
| --- | --- | --- | --- | --- | --- |
| T1 | TR(ms) |  | 11.5 | 2000 |  |
|  | TE(ms) |  | 5 | 2.01 |  |
|  | Slice(mm) | 1 | 1.1 | 1 |  |
| dMRI | TR(ms) | 15763 | 15600 | 3600 |  |
|  | TE(ms) | minimum | 93.4 | 92 |  |
|  | Slice(mm) | 2 | 2.5 | 2 |  |
|  | b-values(s/mm^2^) | 1000 | 1000 | 1000, 2000 |  |
|  | b0 volumes | 5 | 1 | 5 |  |
|  | RPE volumes | 0 | 0 | 3 |  |
|  | Directions | 63 | 25 | 100 |  |
| rs-fMRI | TR(ms) |  |  | 735 | 607 |
|  | TE(ms) |  |  | 39 | 32 |
|  | Slice(mm) |  |  | 2.4 | 2.5 |
|  | Slice number |  |  | 490 | 976 |

Abbreviations: RPE: reverse phase encodings, TR: repetition time, TE: echo time.

## TABLE S2 Demographics of the participants in the datasets used to validate the automatic calculation of DTI-ALPS.

| **Variables** | **MINERVA (n=93)** | **SCANS (n=120)** | **UKB (n=40)** |
| --- | --- | --- | --- |
| Sex (Male), n(%) | 56 (60.2) | 78 (65.0) | 21 (52.5) |
| Age (years), median (IQR) | 70.0 (62.0-78.0) | 71.4 (63.7-76.9) | 64.0 (54.5-70.0) |
| Education (years), median (IQR) | 13.0 (12.0-14.0) | 11.0 (10.0-12.0) | 20.0 (10.0-20.0) |
| WMH volume (mL), median (IQR) | 18.0 (7.1 - 46.0) | 23.9 (13.8-41.2) | 5.3 (0.3 -12.9) |

## TABLE S3 Correlation between manual and automatic calculations of DTI-ALPS, and correlation between the error of automatic method and normalised WMH volume.

| **Dataset** | **Correlation between DTI-ALPS derived from manual vs automatic methods** | **Correlation between error of the automatic method and normalised WMH volume** |
| --- | --- | --- |
| MINERVA | 0.85 *** | 0.14 |
| SCANS | 0.86 *** | 0.05 |
| UKB | 0.89 *** | 0.01 |

P values were represented as: ***: <0.001

## TABLE S4 Demographics of the participants in the datasets used to validate the automatic calculation of BOLD-CSF coupling.

| **Variables** | **ADNI (n=94)** | **UKB (n=100)** |
| --- | --- | --- |
| Sex (Male), n(%) | 39 (41.5) | 52 (52.5) |
| Age (years), median (IQR) | 73.0 (67.0-79.0) | 64.0 (55.0-70.0) |
| Education (years), median (IQR) | 16.0 (14.0-18.0) | 20.0 (10.0-20.0) |

## TABLE S5 Mean dice score of deep learning-segmented CSF compared with the ground truth CSF segmentation at each of the bottom slices in fMRI.

| **Slice number** | **ADNI** | **UKB** |
| --- | --- | --- |
| 1 | 0.677 ±0.143 | 0.656 ±0.180 |
| 2 | 0.641 ±0.131 | 0.620 ±0.184 |
| 3 | 0.621 ±0.129 | 0.609 ±0.175 |
| 4 | 0.598 ±0.158 | 0.613 ±0.168 |

For each slice, if the number of CSF voxels is less than 5, it is excluded from the analysis. **Abbreviations**: ADNI: Alzheimer’s Disease Neuroimaging Initiative, UKB: UK biobank.

## TABLE S6 Correlation between manual and automatic calculations of BOLD-CSF coupling using different numbers of bottom slices.

| **Slices used** | **ADNI** | | **UKB** | |
| --- | --- | --- | --- | --- |
|  | **Available (%)** | **Correlation** | **Available (%)** | **Correlation** |
| Bottom 1 slice | 94.7% | 0.885 | 86.0% | 0.944 |
| Bottom 2 slices | 94.7% | 0.909 | 91.0% | 0.901 |
| Bottom 3 slices | 97.9% | 0.886 | 93.0% | 0.908 |
| Bottom 4 slices | 98.9% | 0.855 | 95.0% | 0.930 |

The “Available (%)” column indicates the percentage of scans with at least 5 CSF-containing voxels in the bottom slices. **Abbreviations**: ADNI: Alzheimer’s Disease Neuroimaging Initiative, UKB: UK biobank.

## TABLE S7 Demographics of the participants in the UK biobank grouped by the availability of each MRI modality.

| **Variables** | **Participants with available data in each MRI modality** | | |
| --- | --- | --- | --- |
|  | T1 (n=41,477) | dMRI (n=39,173) | rs-fMRI (n=37,906) |
| Age (years), median (IQR) | 64.0 (58.0-70.0) | 64.0 (58.0-70.0) | 64.0 (58.0-70.0) |
| Male sex, n (%) | 19438 (46.9) | 18402 (47.0) | 18049 (47.6) |
| Education (years), median (IQR) | 19.0 (10.0-20.0) | 19.0 (10.0-20.0) | 19.0 (10.0-20.0) |
| Follow up, median (IQR) | 5.4 (4.5-6.9) | 5.3 (4.5-6.7) | 5.3 (4.5-6.8) |

**Abbreviations:** dMRI: diffusion MRI, rs-fMRI: resting functional MRI.

## TABLE S8 Correlation between different MRI-based proxies of CSF dynamics.

| **Markers** | **PVS volume** | **DTI-ALPS** | **CP volume** | **BOLD-CSF coupling** |
| --- | --- | --- | --- | --- |
| PVS volume | - | -0.131* | 0.186* | 0.004 |
| DTI-ALPS | -0.131* | - | -0.404* | -0.042* |
| CP volume | 0.186* | -0.404* | - | -0.092* |
| BOLD-CSF coupling | 0.004 | 0.042* | -0.092* | - |

**Abbreviations**: PVS, perivascular space; DTI-ALPS, diffusion tensor image analysis along the perivascular space; BOLD, blood oxygen level dependent; CP, choroid plexus.

*Correlation test p-value < 0.001

## TABLE S9 Sensitivity analysis for MRI-based proxies of CSF dynamics in predicting dementia.

| **Markers** | **Model 1** | | **Model 2** | |
| --- | --- | --- | --- | --- |
|  | HR (CI) | p-value | HR (CI) | p-value |
| PVS volume | 0.979 (0.900-1.064) | 0.610 | 0.987 (0.908-1.072) | 0.750 |
| DTI-ALPS | 0.879 (0.808-0.957) | 0.003 | 0.888 (0.816-0.967) | 0.007 |
| CP volume | 1.182 (1.077-1.298) | <0.001 | 1.143 (1.041-1.256) | 0.007 |
| BOLD-CSF coupling | 0.87 (0.798-0.950) | 0.002 | 0.882 (0.808-0.963) | 0.007 |

Model 1: adjusted for age, sex, education, and median white matter MD

Model 2: adjusted for age, sex, education, median white matter MD, hypertension, diabetes, pack-years of smoking, daily alcohol consumption and APOE4 carrier status

**Abbreviation:** PVS, perivascular space; DTI-ALPS, diffusion tensor image analysis along the perivascular space; CP, choroid plexus; HR, hazard ratio; BOLD-CSF, blood oxygen-level dependent CSF; CI, confidence interval.

## TABLE S10 The association between risk factors and dementia after removing participants whose dementia developed within one year of MRI visit.

| **Variables** | **HR (95% CI)** | **p-value** |
| --- | --- | --- |
| **Cardiovascular risk factors** | | |
| SBP | 0.954 (0.873-1.042) | 0.354 |
| DBP | 0.984 (0.905-1.069) | 0.690 |
| Pulse pressure | 0.954 (0.869-1.046) | 0.354 |
| Duration of hypertension (years) | 1.142 (1.089-1.199) | <0.001* |
| Diabetes | 1.118 (1.06-1.178) | <0.001* |
| Duration of diabetes (years) | 1.060 (1.024-1.097) | 0.002* |
| Smoking | 1.212 (1.158-1.268) | <0.001* |
| Alcohol | 1.047 (0.969-1.132) | 0.354 |
| **Cardiac and arterial function markers** | | |
| LV ejection fraction | 0.861 (0.807-0.918) | <0.001* |
| Arterial stiffness | 1.016 (0.999-1.035) | 0.064 |
| Carotid IMT | 1.095 (1.023-1.172) | 0.011* |
| **Imaging marker** | | |
| WMH volume | 1.117 (1.059-1.179) | <0.001* |
| PVS volume | 1.013 (0.94-1.091) | 0.740 |
| DTI-ALPS | 0.866 (0.796-0.942) | 0.001* |
| CP volume | 1.185 (1.088-1.291) | <0.001* |
| BOLD-CSF coupling | 0.875 (0.806-0.951) | 0.001* |

All analyses were adjusted for age, sex and years of education.

**Abbreviation:** SBP, systolic blood pressure; DBP, diastolic blood pressure; LV, left ventricle; IMT, intima-media thickness; WMH, white matter hyperintensity

*: p<0.05 after Benjamini-Hochberg correction

## TABLE S11 The association between risk factors and dementia

| **Variables** | **HR(CI)** | **p-value** | **Schonfield residual test p-value** |
| --- | --- | --- | --- |
| **Cardiovascular risk factors** |  |  |  |
| SBP | 0.953 (0.873-1.041) | 0.343 | 0.961 |
| DBP | 0.983 (0.904-1.069) | 0.690 | 0.966 |
| Pulse pressure | 0.953 (0.869-1.046) | 0.343 | 0.937 |
| Duration of hypertension (years) | 1.142 (1.089-1.199) | <0.001* | 0.417 |
| Diabetes | 1.118 (1.060-1.178) | <0.001* | 0.937 |
| Duration of diabetes (years) | 1.060 (1.024-1.097) | 0.002* | 0.885 |
| Smoking | 1.212 (1.158-1.268) | <0.001* | 0.966 |
| Alcohol | 1.047 (0.969-1.132) | 0.343 | 0.664 |
| **Cardiac and arterial function markers** |  |  |  |
| LV ejection fraction | 0.861 (0.807-0.918) | <0.001* | 0.966 |
| Arterial stiffness | 1.017 (0.999-1.035) | 0.063 | 0.664 |
| Carotid IMT | 1.095 (1.023-1.172) | 0.012 | 0.885 |
| **CSVD imaging marker** |  |  |  |
| WMH volume | 1.117 (1.059-1.179) | <0.001* | 0.966 |

All analyses were adjusted for age, sex and years of education. Schonfield residual test p-value greater than 0.05 suggests the validity of the proportional hazard model.

**Abbreviation**: SBP, systolic blood pressure; DBP, diastolic blood pressure; LV, left ventricle; IMT, intima-media thickness; WMH, white matter hyperintensity

*: p<0.05 after Benjamini-Hochberg correction

## TABLE S12 The mediation effect of DTI-ALPS between WMH and dementia after correcting for median WM MD

| **Variables** | **Effect** | **HR (95% CI)** | **p-value** | **%mediation** |
| --- | --- | --- | --- | --- |
| WMH volume | Indirect | 1.064 (1.023-1.117) | <0.001* | 34.6% |
|  | Direct | 1.137 (1.008-1.217) | <0.001* |  |
|  | Total | 1.209 (1.065-1.295) | <0.001* |  |

All analysis was corrected for age, sex, education, and median WM MD.

**Abbreviation:** DTI-ALPS, diffusion tensor image analysis along the perivascular space; HR, hazard ratio; CI, confidence interval.

*: p<0.05 after Benjamini-Hochberg correction

# Supplementary Figures

## FIGURE S1 Cohort selection.

**
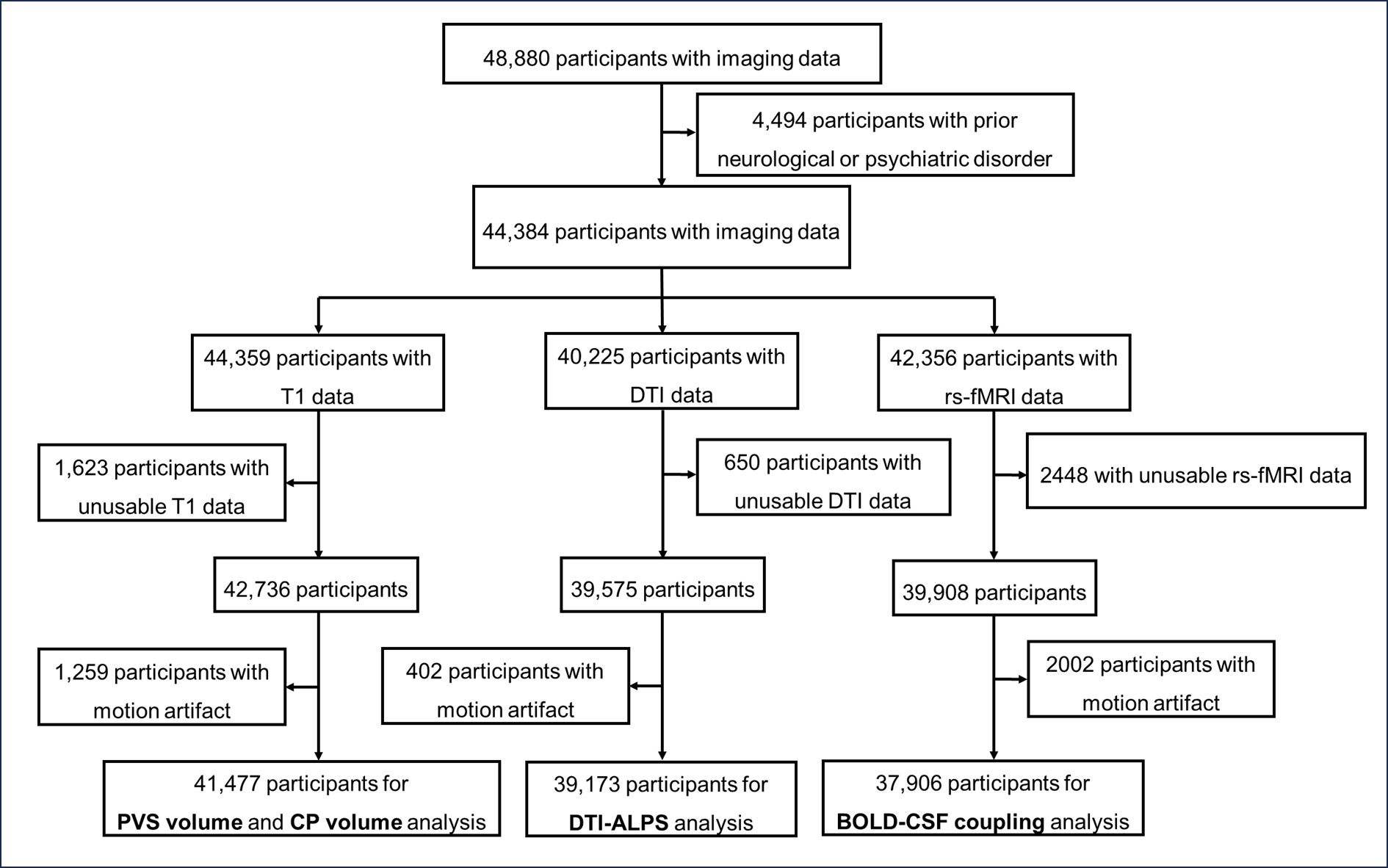
**

## FIGURE S2 Examples of ROI placement in DTI-ALPS calculation in UKB cohort.

**
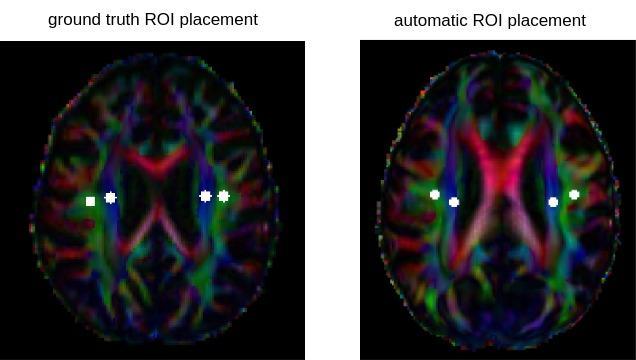
**

## FIGURE S3 Correlation between manual and automatic calculations of DTI-ALPS in different datasets.


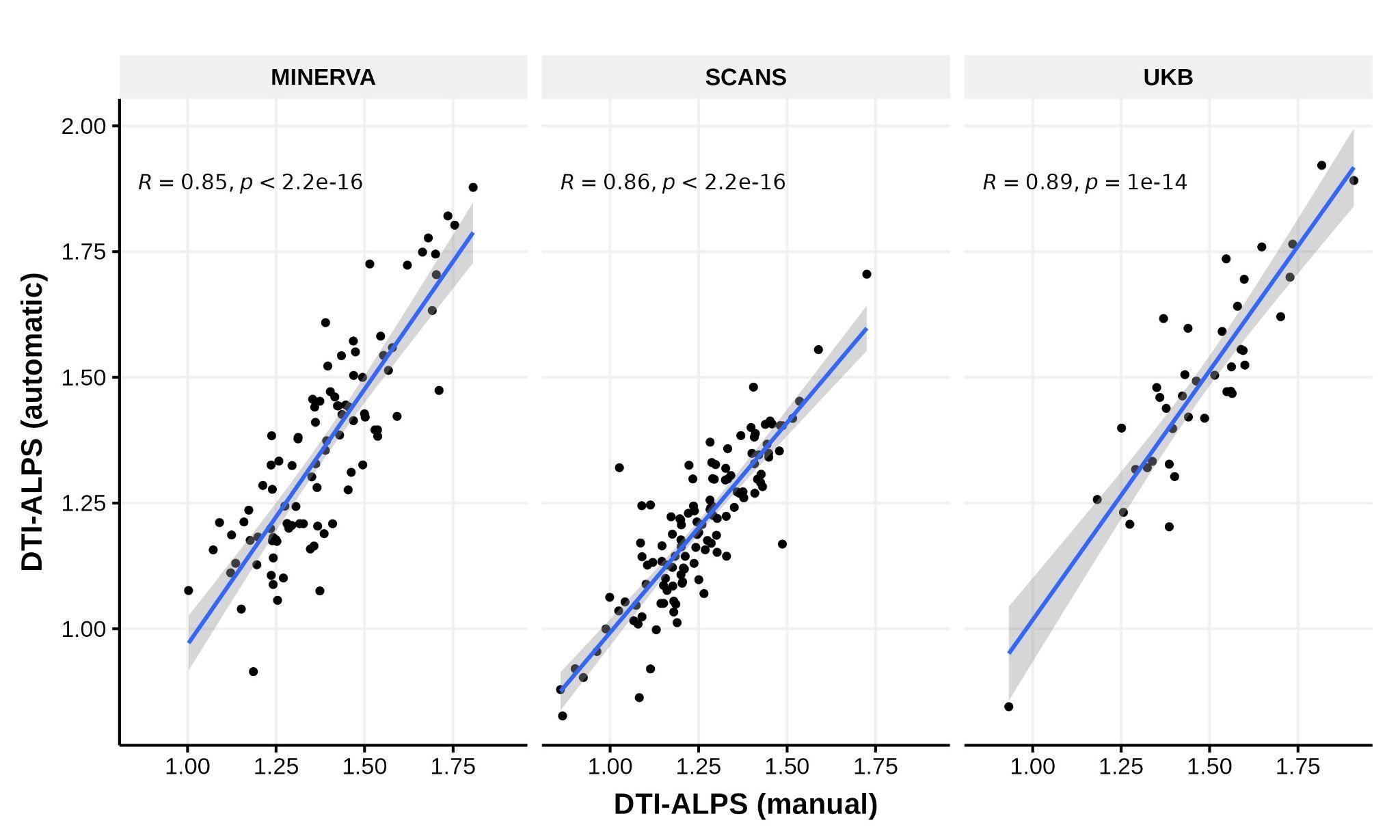


## FIGURE S4 BOLD -CSF coupling algorithm.


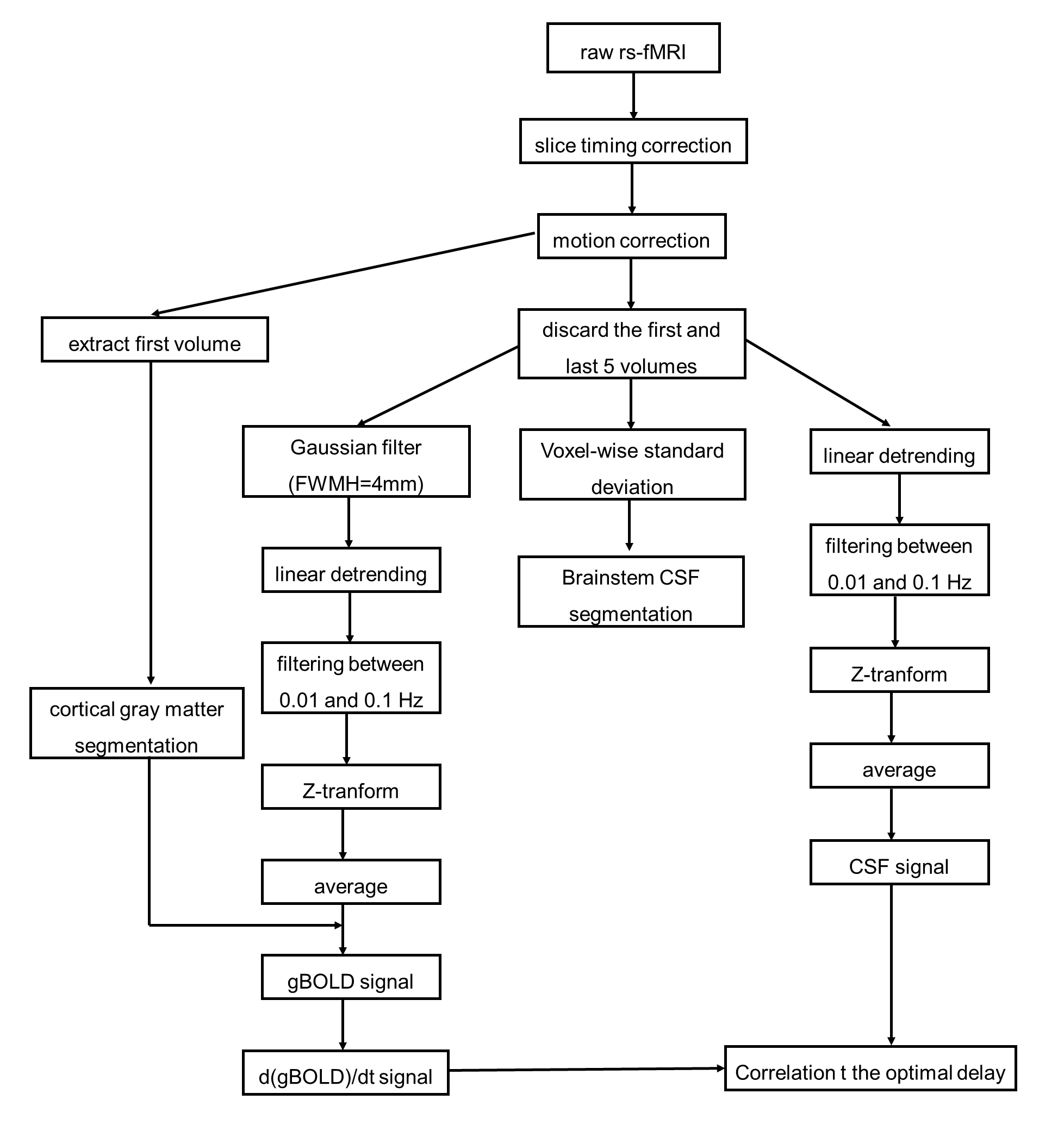


**Abbreviations:** FWHM: full width half maximum, gBOLD: global blood oxygen level dependent, d(gBOLD)/dt: first derivative of the gBOLD signal with respect to time, CSF: cerebrospinal fluid

## FIGURE S5 One example of CSF segmentation (red region) using FreeSurfer on fMRI.


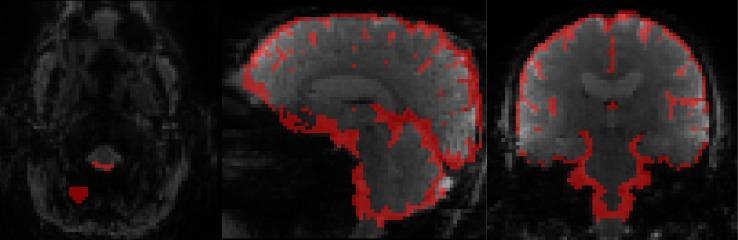


## FIGURE S6 Examples of CSF segmentation from ADNI and UKB. f0 image: the first volume in fMRI.


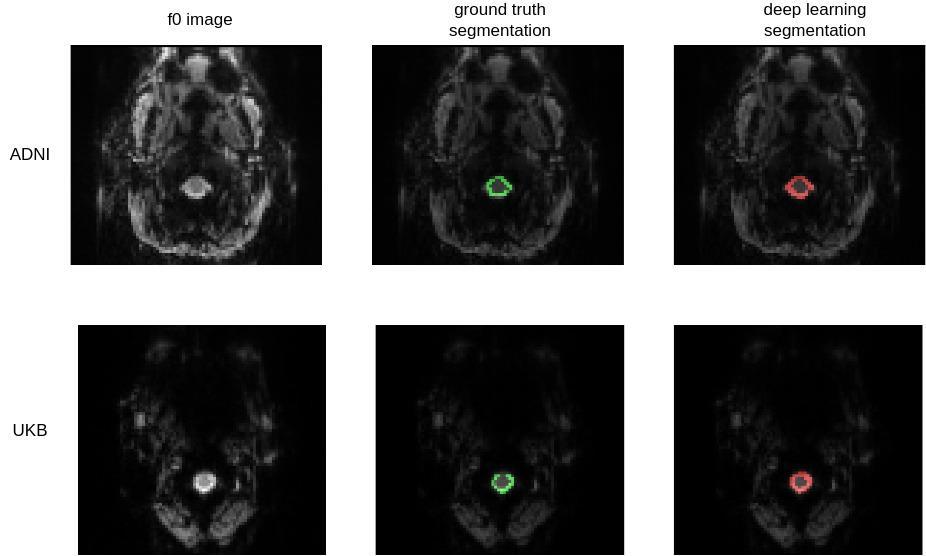


## FIGURE S7 MRI-based proxies of CSF dynamics mediate the association between cardiovascular functions and WMH.


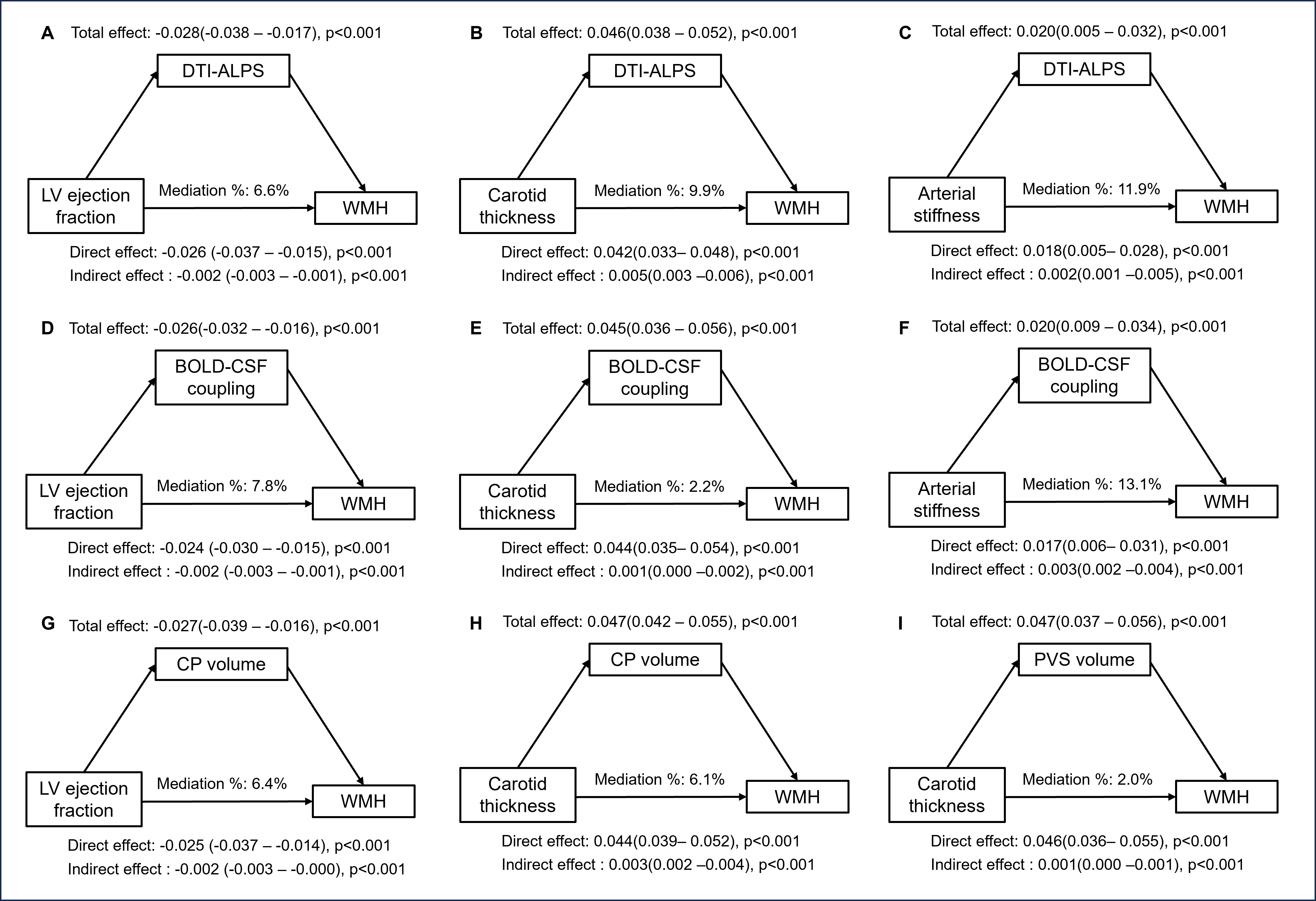


**Abbreviations:** LV: left ventricle, WMH: white matter hyperintensity. DTI-ALPS, diffusion tensor image analysis along the perivascular space; BOLD, blood oxygen level dependent; CP, choroid plexus.

# References

[1] Brown RB, Tozer DJ, Loubière L, Hong YT, Fryer TD, Williams GB, et al. MINocyclinE to Reduce inflammation and blood brain barrier leakage in small Vessel diseAse (MINERVA) trial study protocol. European stroke journal. 2022;7:323-30.

[2] Walsh J, Tozer DJ, Sari H, Hong YT, Drazyk A, Williams G, et al. Microglial activation and blood-brain barrier permeability in cerebral small vessel disease. Brain : a journal of neurology. 2021;144:1361-71.

[3] Cedres N, Ferreira D, Machado A, Shams S, Sacuiu S, Waern M, et al. Predicting Fazekas scores from automatic segmentations of white matter signal abnormalities. Aging (Albany NY). 2020;12:894-901.

[4] Zeestraten EA, Lawrence AJ, Lambert C, Benjamin P, Brookes RL, Mackinnon AD, et al. Change in multimodal MRI markers predicts dementia risk in cerebral small vessel disease. Neurology. 2017;89:1869-76.

[5] Liu X, Barisano G, Shao X, Jann K, Ringman JM, Lu H, et al. Cross-Vendor Test-Retest Validation of Diffusion Tensor Image Analysis along the Perivascular Space (DTI-ALPS) for Evaluating Glymphatic System Function. Aging and disease. 2024;15:1885-98.

[6] Mojiri Forooshani P, Biparva M, Ntiri EE, Ramirez J, Boone L, Holmes MF, et al. Deep Bayesian networks for uncertainty estimation and adversarial resistance of white matter hyperintensity segmentation. Human brain mapping. 2022;43:2089-108.

[7] Billot B, Greve DN, Puonti O, Thielscher A, Van Leemput K, Fischl B, et al. SynthSeg: Segmentation of brain MRI scans of any contrast and resolution without retraining. Medical image analysis. 2023;86:102789.

[8] Fultz NE, Bonmassar G, Setsompop K, Stickgold RA, Rosen BR, Polimeni JR, et al. Coupled electrophysiological, hemodynamic, and cerebrospinal fluid oscillations in human sleep. Science (New York, NY). 2019;366:628-31.

[9] Jack CR, Jr., Arani A, Borowski BJ, Cash DM, Crawford K, Das SR, et al. Overview of ADNI MRI. Alzheimers Dement. 2024;20:7350-60.
